# An Unfortunate Cause of Chronic Nausea and Vomiting: Mitochondrial Neurogastrointestinal Encephalomyopathy (MNGIE)

**DOI:** 10.1155/2022/7398292

**Published:** 2022-03-30

**Authors:** Neethi Dasu, Brian Blair, C. Jonathan Foster, Colin Smith

**Affiliations:** ^1^Department of Gastroenterology, Jefferson Health New Jersey, Cherry Hill, NJ, USA; ^2^Department of Gastroenterology, Jefferson Medical College of Thomas Jefferson University, Philadelphia, PA, USA

## Abstract

We present a unique case of a 24-year-old male who was admitted for intractable nausea, emesis, weight loss, and abdominal discomfort. The patient underwent an extensive workup and was diagnosed with mitochondrial neurogastrointestinal encephalopathy. Early diagnosis is critical to proper management of this disease process. MGNIE is a difficult disorder to diagnose given the complexity of the disease, and this case provides clinicians the proper understanding and management of such a unique and difficult diagnosis.

## 1. Introduction

Mitochondrial neurogastrointestinal encephalomyopathy (MNGIE) is a progressive, autosomal recessive, degenerative disease which portends significant morbidity and mortality [1]. It is an extremely rare and devastating disease with a prevalence of 1–9 in 1000000 worldwide [1]. The average age of onset is 18, and the disease usually manifests in the first two decades of life [1]. Mutations in the TYMP gene lead to loss of function in thymidine phosphorylase activity which causes MNGIE [1, 2]. MNGIE is often misdiagnosed given the complexity in its presentation and subtle nonspecific symptoms. We present a case of a young male who was initially presented with chronic symptoms of malnutrition, nausea, and emesis, and he was subsequently diagnosed with MNGIE.

## 2. Case Report

**Figure 1:**
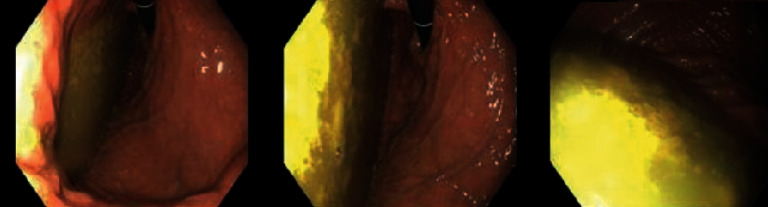
EGD with food in the stomach consistent with gastric atony.

A 24-year-old male with a medical history of anorexia, depression, clostridium difficile infection, weight loss, nausea, vomiting, diarrhea, and abdominal pain presented for evaluation of severe malnutrition and intractable nausea that had been occurring intermittently for three years as well as intermittent diarrhea and weight loss of seventy pounds. The patient was initially lost to follow-up in the outpatient setting and had underwent bidirectional endoscopy two years prior which revealed pangastritis with food in the stomach (Figure 1). Biopsies were negative for intestinal metaplasia, infiltrative disease, celiac disease, lymphoma, *H. pylori*, or inflammatory bowel disease. Ascending colon and rectum biopsies were positive for chronic inflammation and reactive changes.

**Figure 2:**
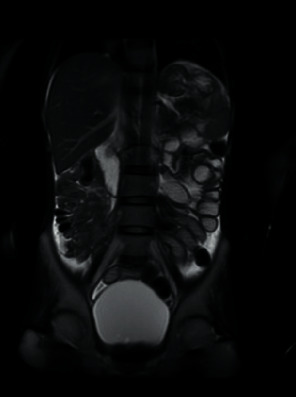
MRE showing diffuse ileus.

On a physical exam, the patient was cachectic with bitemporal and diffuse muscle wasting, flexion contracture of left 5th metacarpal, and poor dentition. Initial lab work revealed the following: lactate of 6.8 (mg/dL), alkaline phosphatase of 198 (IU/L), aminotransferase of 42 (IU/L), alanine transferase of 58 (IU/L), prealbumin 12 (mg/dL), c-reactive protein of 27 (mg/L), gastrin of 67 (pg/mL), fecal calprotectin of 296 (*µ*g/g), lactoferrin 313.40 (*μ*g/g), and elastase (*μ*g/g), within normal limits. Magnetic resonance enterography (MRE) was consistent with diffuse ileus (Figure 2). Due to his intractable symptoms, the patient underwent insertion of a gastrojejunostomy (GJ) tube and was started on tube feeds. A gastric emptying study was positive for delayed gastric emptying, and he was treated with metoclopramide and underwent esophagogastroduodenoscopy (EGD) with botulinum injection; stomach biopsies revealed a mild increase in intraepithelial T cells.

The patient's hospital course was complicated by aspiration pneumonia and malfunctioning of his GJ tube which required revision. Due to persistent elevated lactate levels, motility workup was ordered which included anticentromere antibody, aldolase levels, myositis specific antibodies, amino acid panel, RNA polymerase 3 antibody, scleroderma 70 antibody, thymidine phosphorylase (TP) enzyme analysis, plasma thymidine, and deoxyuridine analyte. The patient left against medical advice and was admitted to another hospital for aspiration pneumonia requiring intubation and eventual tracheostomy placement. The motility workup was negative except for thymidine and deoxyuridine analytes were noted to be elevated, revealing a thymidine phosphorylase deficiency, and the patient was diagnosed with MNGIE.

## 3. Discussion

MNGIE is a deadly mitochondrial multisystem disorder caused by mutations in the TYMP gene which leads to deficiency of thymidine phosphorylase activity [1, 2]. The TYMP gene is responsible for the degradation of thymidine and its dysfunction leads to a cascade of events including compromised mitochondrial DNA repair, loss of oxidative phosphorylation, nucleoside aggregation, and subsequent compounding of mutations [1, 2]. The TYMP gene is readily found throughout the human body, especially in the gastrointestinal tract, brain, blood, and nervous system. Epidemiologically, the prevalence of this disorder is less than ten in one million [1, 3]. The majority of individuals begin experiencing symptoms before the age of twenty with the mean age at onset being approximately 18 years [3]. MNGIE is distributed through multiple ethnic populations and is inherited in an autosomal recessive fashion [4].

The major clinical characteristics of MNGIE include progressive gastrointestinal dysmotility, leukoencephalopathy, peripheral neuropathy, and ocular manifestations [1, 3]. Gastrointestinal (GI) symptoms can present as diarrhea, nausea, emesis, cramping, gastroparesis and can lead to severe malnutrition and cachexia [1, 3]. Our patient presented with significant weight loss, malnutrition, postprandial abdominal discomfort, nausea, and emesis as well as delayed gastric emptying; these symptoms were consistent with the major GI characteristics of MNGIE; however, due to their nonspecificity, the diagnosis was somewhat delayed. Manifestations of leukoencephalopathy and peripheral neuropathy were also seen in our patient as he was profoundly weak and had contractures and progressive muscle wasting. Furthermore, he was diagnosed with depression multiple years ago which is a neurologic manifestation of MNGIE.

The clinical diagnosis of MNGIE disease is based on the presence of the major clinical characteristics or by detection of pathogenic mutations in TYMP, reduced levels of thymidine phosphorylase enzyme activity, or elevated plasma concentrations of deoxyuridine and thymidine [3]. In this case, plasma level of thymidine was noted to be 15.83 (normal <0.25 *μ*mol/L) and deoxyuridine was 25.92 (normal <0.25 *μ*mol/L), which was consistent with MNGIE. Some patients might have elevations in serum lactate which was a diagnostic clue in this case as the patient was noted to have persistently elevated lactate levels for months.

Treatment options are limited and based on symptom management including analgesics, antiemetics, probowel motility stimulant drugs, as well as treatment of malnutrition with parenteral nutrition or total parenteral nutrition [1]. Since many patients suffer from nausea, vomiting, weight loss, global dysmotility, and cachexia, treatment includes nutritional support, gastrostomy tube placement, and antibiotic treatment for bacterial overgrowth [5]. Medication regimens include domperidone for nausea and vomiting, as well as morphine, gabapentin, tricyclic antidepressants, and anticonvulsant medicines for relief of symptoms. Physical therapy and occupational therapy may help increase and preserve mobility [1, 5].

Drugs which are hepatically metabolized or that affect the mitochondrial metabolism should be avoided [3]. Drugs such as valproate, phenytoin, chloramphenicol, tetracycline, and antipsychotic medications can interfere with mitochondrial function and should also be avoided [5].

Currently, there are investigational therapies which include enzyme supplementation, stem cell transplantation (HPSCT), hemodialysis to decrease toxic metabolites, and liver transplantation [1, 6]. Liver transplant has been shown to be beneficial in certain patients. TP enzyme activity in the donor graft was sufficient to lower plasma thymidine to near-normal levels [7]. Posttransplant plasma thymidine levels were noted to be diminished in certain populations, with one study demonstrating that thymidine levels dropped more than 20 times lower than pretransplant levels with noted improvement in motility issues and neurologic deficits [7]. HPSCT can also be used to treat patients, and however, this treatment has a high mortality rate.

Gene therapy is currently experimental with promising murine studies. One study which utilized using viral DNA to deliver the TYMP gene to the liver reduced thymidine and deoxyuridine levels to normal levels in about half of the mice studied, showing potential that gene therapy can treat MNGIE [8].

As the disease progresses, patients die from GI complications, malnutrition, or infections, and the average age of death is 37 years [1]. Our patient chose to pursue hospice and died a few months later. Early diagnosis requires a high index of suspicion and recognition of symptoms before tissue damage and cachexia occurs. Treatment efficacy was higher in patients who did not have severe intestinal and peripheral nerve damage [9].

In conclusion, this case is important because it highlights a rare cause of common gastrointestinal symptoms. Most patients are often misdiagnosed due to the subtlety and nonspecificity of symptoms; thus, this case further emphasizes that gastroenterologists should be vigilant in recognizing rare disorders associated with GI dysmotility, especially in young patients. MNGIE should be suspected in all patients who present with both nervous system and gastrointestinal symptoms [10].

## Data Availability

No data were used to support this study.
